# Effectiveness of a 4-day intensive course for neurosurgeons in error reduction in microvascular anastomoses

**DOI:** 10.1016/j.bas.2025.105623

**Published:** 2025-10-01

**Authors:** Teona Z. Carciumaru, Victor Eşanu, Cadey Tang, George C. Dindelegan, Nikolay Velinov, Clemens Dirven, Dalibor Vasilic, Ruben Dammers, Torstein R. Meling, Victor Volovici

**Affiliations:** a Department of Neurosurgery, Erasmus MC University Medical Center, Rotterdam, the Netherlands; b Department of Plastic and Reconstructive Surgery, Erasmus MC University Medical Center, Rotterdam, the Netherlands; c Center for Complex Microvascular Surgery, Erasmus MC University Medical Center, Rotterdam, the Netherlands; d Department of General Surgery, “Iuliu Hatieganu” University of Medicine and Pharmacy, Cluj-Napoca, Romania; e Department of Experimental Microsurgery, Simulation and Experiment Center, “Iuliu Hatieganu” University of Medicine and Pharmacy, Cluj-Napoca, Romania; f Clinics of Neurosurgery, Vascular and Endovascular Neurosurgery, University Hospital Pirogov, Sofia, Bulgaria; g Department of Neurosurgery, Rigshospitalet University Hospital, Copenhagen, Denmark

**Keywords:** Microvascular anastomosis, Neurovascular training, Surgical education, Surgical skill acquisition, Microsurgical assessment

## Abstract

**Introduction:**

Microvascular anastomosis is a critical but technically demanding skill in neurosurgery, with a long learning curve and high potential for error. Intensive hands-on training, such as that provided by the European Association of Neurosurgical Societies (EANS), aims to improve surgical proficiency, but objective evaluations of such courses are limited.

**Research question:**

Does participation in the EANS Step I and Step II microvascular training courses lead to a significant reduction in technical errors during microvascular anastomosis?

**Material and methods:**

A pre- and post-intervention study design was used to assess error reduction in participants of the EANS microvascular courses. The 92 participants were evaluated on a chicken end-to-end arterial anastomosis on the first and last days of course by two independent, blinded assessors using the Anastomosis Lapse Index (ALI).

**Results:**

In the Step I cohort, the overall median error count decreased by 32.5 % (from 20 to 13.5, P < 0.0001). In the Step II cohort, the reduction was 46.9 % (from 16 to 8.5, P < 0.0001). Back-wall stitches, a critical error, was significantly reduced in both cohorts (P < 0.0001).

**Discussion and conclusion:**

Participation in the Step I and Step II EANS courses significantly improved microsurgical performance, as evidenced by reduced technical errors in microvascular anastomosis. Future efforts should focus on refining evaluation tools and on integrating technological innovations such as motion tracking and machine learning for enhanced feedback and long-term skill assessment.

## Highlights

• Hands-on EANS courses improve microvascular anastomosis performance
• Step I course lowered median errors by 32.5 %
• Step II course lowered median errors by 46.9 %
• Critical errors, like back-wall stitches, were reduced post-training
• Results support short, intensive microsurgical training in neurosurgery

## 1 Introduction

Microvascular anastomosis is a complex and essential technique in neurosurgery. It requires a high degree of precision, dexterity, and thorough understanding of vascular anatomy (Byvaltsev et al., 2018). The long learning curve associated with mastering this technique often spans several years of training (Khachatryan et al., 2021; Younus et al., 2021), and errors can have severe clinical consequences, such as compromised vascular flow and postoperative stroke (Powers et al., 2011). Despite its importance, the number of practitioners with a high degree of proficiency in microvascular anastomoses in neurosurgery remains limited due to the high level of specialization required and the increasing prevalence of endovascular techniques (Zaed et al., 2019).

To address these challenges, specialised training programs for neurosurgeons and residents have been introduced to supplement traditional education. However, many of these lack objective assessments of their effectiveness. The European Association of Neurosurgical Societies (EANS) offers 4-day intensive courses designed to improve microvascular anastomosis skills through hands-on practice under the guidance of experienced mentors. This concentrated course provides participants with the opportunity to develop technical skills in a controlled environment, potentially shortening the learning curve and reducing errors during microvascular procedures.

This study aims to evaluate the effectiveness of the EANS courses in reducing errors during microvascular anastomosis. We hypothesise that participation in the course will lead to a substantial reduction in errors made in post-course evaluations compared to pre-course performance. By investigating the impact of this intensive training program, this research will provide insights into the role of short-duration, high-intensity courses in improving skill acquisition and error reduction in neurosurgical practice.

## 2 Methods

### 2.1 Study design

This study employed a pre- and post-intervention design to evaluate the effectiveness of the EANS Advanced Microvascular Hands-On Course in reducing errors during microvascular anastomosis. Participants provided informed consent prior to their inclusion in the study. The medical ethics committee of the “Iuliu Hațieganu” University of Medicine and Pharmacy waived the need for informed consent for the participants and approved the study protocol. Nevertheless, all participants were required to sign consent forms and agreed to the collection of anonymized data.

The course was modelled after a 10-day microsurgical comprehensive course lead by the senior author (VV), which has been running for over 30 years in Cluj-Napoca, Romania, training several hundreds of final year residents and fellows of different specialties. The techniques employed during this original course ranged from very basic exercises to heterotopic transplant models in the last days. Nevertheless, especially in the time frame 2014–2018, the course was made more efficient through research efforts aimed at identifying exercises most crucial to skill acquisition (Dindelegan et al., 2021; Orădan et al., 2021; Eşanu et al., 2022; Volovici et al., 2021). Therefore, after a thorough brainstorm session between the senior authors (TRM and VV), the decision was made to model the course around maximizing performance around end-to-end and end-to-side anastomoses by providing models that offered the steepest, most effective learning curve, from the chicken leg femoral anastomosis to the femoral vein anastomosis in live rats. The first editions of the course (2018–2022) benefitted from this “Step I″ design, aimed at participants with minimal microsurgical experience. In 2022, based on popular demand by the course participants, the Advanced course (Step II) was set up. The advanced course focused on variations of the end-to-side anastomosis, side-to-side anastomosis, and calibre mismatch. It featured only complex microsurgical models and expected participants to have a solid grasp of microsurgical techniques. In the last day, bypass in the deep field was the main practice exercise. While the course begins with 3 h training on the chicken leg arteries, it continues with 3.5 days of live animal training only, with exercises ranging from the rat aorta end-to-end to complex interposition grafts and bypasses in the last day, including deep field anastomosis with 3D printed skulls, made to simulate various approaches.

Participants were tasked with an end-to-end anastomosis of a 2.1–2.5 mm diameter chicken ischiatic artery, a validated microsurgical training model (Dumestre and YeungClaireTemple-Oberle, 2014). They were evaluated at the end of the first and last day of each course by two independent blinded faculty members with extensive microsurgical experience (NV and RD). The assessment was conducted using the Anastomosis Lapse Index (ALI) score describing 10 possible errors (Ghanem et al., 2016), and averaged scores were used for analysis.

### 2.2 Study population and setting

Participants of neurosurgical background, who attended either the Step I EANS Microneurosurgery Hands-On Course or the Step II EANS Advanced Microvascular Hands-On Course, were included. The courses were designed to provide intensive, hands-on training in microvascular anastomosis. Each course included practical sessions under the supervision of experienced mentors who provided live demonstrations and structured individualised feedback throughout training. The curriculum covered essential techniques and provided opportunities for participants to practice and refine their skills in a controlled environment.

Four Step I and one Step II courses took place in the study, summing a total of 92 participants. The Step I courses contained 71 participants, and the Step II course contained 21 participants. One Step I course, which included 21 participants, was excluded from the main analysis because the data recorded by one of the assessors only indicated the presence of a certain error type, without quantifying the number of errors. As the ALI score is a frequency-based score, this data was not useable, and the final analysis included 50 Step I participants and 21 Step II participants (total n = 71).

### 2.3 Statistical analysis

The distribution of the data was tested for normality using the Shapiro-Wilk test which showed no normal distribution, except for post-course anastomosis scores of the EANS Microneurosurgery Hands-On Course. Therefore, we chose to describe participants' performance metrics before and after the course using medians and interquartile ranges (IQRs). The Wilcoxon signed-rank test tested the difference in medians before and after the course, with statistical significance set at *P* < 0.005 after applying a Bonferroni correction for multiple comparisons. Interrater reliability was assessed using weighted Cohen's kappa and the Intraclass Correlation Coefficient (ICC).

## 3 Results

### 3.1 Step I EANS Microneurosurgery Hands-On Course specific results

**Fig. 1:**
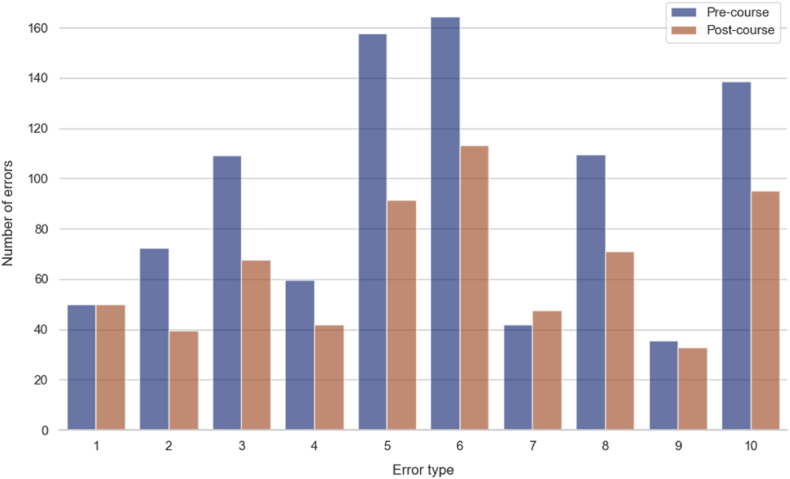
Change in the total number of errors from all participants before and after the Step I EANS Microneurosurgery Hands-On Course.

**Fig. 2:**
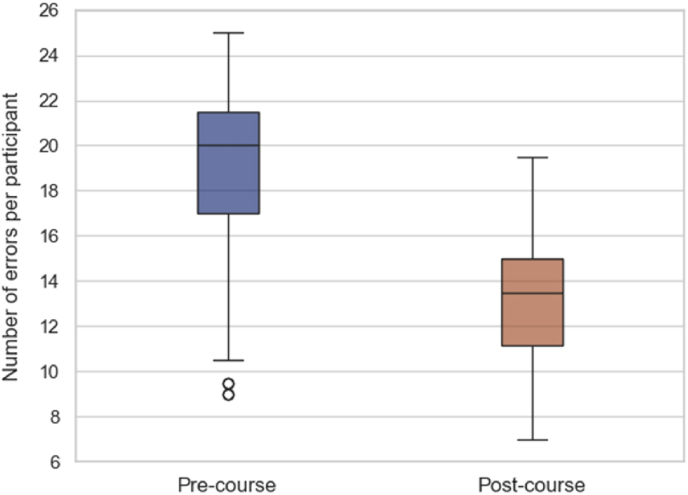
Change in total number of errors per participant before and after the Step I EANS Microneurosurgery Hands-On Course.

The participants' error counts were analysed before (pre-course, day 1) and after (post-course, day 4) the Step I course. The findings are summarized in Table 1, which presents the median error counts and interquartile ranges (IQR) for various error types, along with the p-values indicating the statistical significance of the changes. The total pre-course and post-course error counts are seen in Fig. 1. The overall median error count for all participants significantly decreased by 32.5 %, from 20 (IQR: 17–21.5) to 13.5 (IQR: 11.13–15, *P* < 0.0001), as seen in Fig. 2.

**Table 1:** Outcomes before and after the step I EANS microneurosurgery hands-on course.

Error Type	Pre-course Median (IQR)	Post-course Median (IQR)	*P-*value
1	1 (1–1)	1 (1–1)	–
2	1.5 (1–2)	1 (0.5–1)	**< 0.0001**
3	2.5 (1.63–3)	1.5 (1–2)	**< 0.0001**
4	1 (1–1.5)	1 (0.5–1)	**0.0002**
5	3.5 (2.5–4)	2 (1–2.5)	**< 0.0001**
6	3.5 (2.63–4)	2 (2–2.5)	**< 0.0001**
7	1 (1–1)	1 (1–1)	0.056
8	2.5 (2–2.5)	1.25 (1–2)	**< 0.0001**
9	1 (0.5–1)	0.75 (0.5–1)	0.49
10	3 (2–3.5)	2 (1.5–2.5)	**< 0.0001**
Overall	20 (17–21.5)	13.5 (11.13–15)	**< 0.0001**

Bold data indicates statistically significant.

Error type 2 is particularly critical to avoid, as it involves inadvertently catching the back- or sidewall when taking suture bites, which can cause occlusion of the lumen and failure of the anastomosis. The data shows a significant reduction in the median error count for this error, from 1.5 (IQR: 1–2) before the course to 1 (IQR: 0.5–1) after the course *(P* < 0.001).

The median error counts for error types 1 and 7 remained unchanged before and after the course. Error type 9 did not show a statistically significant difference, with values changing from 1 (IQR: 0.5–1) to 0.75 (IQR: 0.5–1) (*P* = 0.49). All other error types were significantly reduced, as detailed in Table 1.

### 3.2 Step II EANS Advanced Microvascular Hands-On Course specific results

**Fig. 3:**
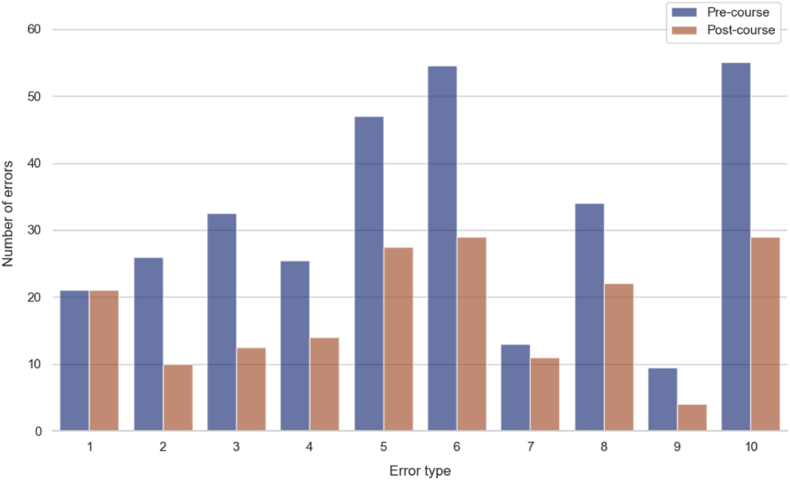
Change in total number of errors from all participants before and after the Step II EANS Advanced Microvascular Hands-On Course.

**Fig. 4:**
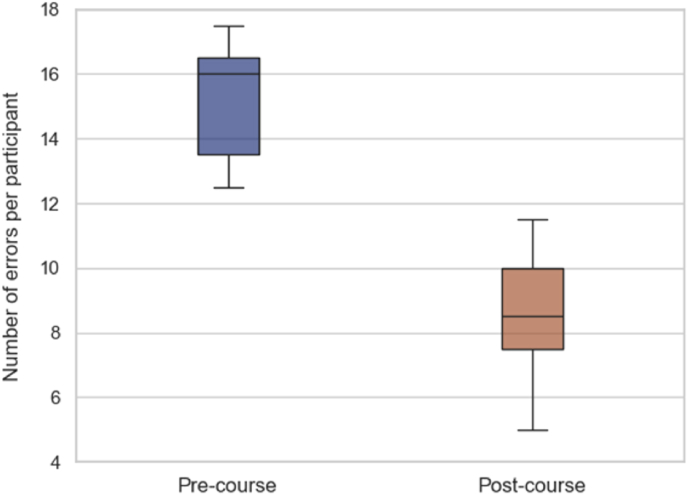
Change in total number of errors per participant before and after the Step II EANS Advanced Microvascular Hands-On Course.

The participants' error counts were analysed before (pre-course, day 1) and after (post-course, day 4) the Step II course. Table 2 summarizes the median error counts and interquartile ranges (IQR) for various error types, along with p-values indicating statistical significance. Fig. 3 shows the total pre-course and post-course error counts. The overall median error count significantly decreased by 46.9 %, from 16 (IQR: 13.5–16.5) to 8.5 (IQR: 7.5–10, *P* < 0.0001), as shown in Fig. 4.

**Table 2:** Outcomes before and after the step II EANS advanced microvascular hands-on course.

Error Type	Pre-course Median (IQR)	Post-course Median (IQR)	*P-*value
1	1 (1–1)	1 (1–1)	–
2	1 (1–1.5)	0.5 (0–1)	**0.0002**
3	1.5 (1–2)	0.5 (0–1)	**0.0004**
4	1 (1–1.5)	1 (0–1)	0.0008
5	2 (2–3)	1 (1–1.5)	**0.0002**
6	2.5 (2–3)	1.5 (1–2)	**< 0.0001**
7	0.5 (0.5–1)	0.5 (0–1)	0.21
8	1.5 (1–2)	1 (1–1.5)	**0.0013**
9	0.5 (0–1)	0 (0–0)	0.022
10	2.5 (2.5–3)	1.5 (1–1.5)	**< 0.0001**
Overall	16 (13.5–16.5)	8.5 (7.5–10)	**< 0.0001**

Bold data indicates statistically significant.

Once again, error type 2 shows a significant reduction in the median error count, decreasing from 1 (IQR: 1–1.5) before the course to 0.5 (IQR: 0–1) after the course (*P* < 0.0001). Apart from errors 1, 4, 7 and 9, all other error types show a significant reduction in occurrence (Table 2).

### 3.3 Interrater reliability

The interrater reliability assessment yielded a weighted Cohen's kappa was 0.79 and an ICC of 0.71 (95 % CI: 0.62–0.78). These values reflect consistent interrater agreement.

## 4 Discussion

Our study assessed whether the EANS courses for microneurosurgery improve microvascular anastomoses evaluated with the validated ALI score. There was a significant reduction in most error types, with the median overall score improving by 32.5 % and 46.9 % within the respective cohorts *(P* < 0.001). While few studies have specifically assessed the impact of microsurgical training using the ALI score, Paladino et al. (2021) reported a significant overall error reduction following expert-led training on a rat model. Their analysis included completion time and patency but did not examine changes in specific error types. By analysing the changes in specific errors, we provide a detailed understanding of how targeted training affects different aspects of performance. The nonsignificant changes in errors 7 (vessel wall tear) and 9 (thread in lumen) may reflect their low baseline frequency, whereas error 1 (interrupted anastomosis line) is influenced by its dependence on other errors. Of particular importance is the reduction in errors most likely to compromise an anastomosis even if no other errors are present, such as error 2 (back wall stitch). The design of training programs that minimise such errors is important for microsurgical training, and future iterations could also incorporate structured participant feedback.

The ALI score is a practical and objective tool for assessing microvascular anastomosis skills and has been widely adopted in training settings (Gholami et al., 2024). While its simplicity is a strength, it does not currently incorporate factors such as time to completion or live model outcomes, both of which are important for clinical performance. As a result, our study did not test patency or completion time, which limits the transferability of results to clinical contexts. The score's equal weighting of all error types, regardless of clinical severity, may also reduce its discriminatory power. While the ALI score remains a practical and accessible metric, further refinement is warranted to increase its clinical relevance. Future studies should also explore whether improvement in evaluation score translates to increased clinical performance.

A further consideration relates to the timeframe of assessment. Our evaluation was confined to immediate post-training performance, without assessment of long-term skill retention. Longitudinal evaluations provide insight into how skills are consolidated and sustained over time. A recent review reported that structured, long-term training was among the most influential factors in microsurgical performance (Molle and La Padula, 2024). Variability in baseline skill levels among participants must also be considered, as they are difficult to quantify yet possibly influence outcomes (Volovici and Dindelegan, 2021). Although errors were recorded at day 1, prior experience and innate learning potential were not accounted for. Future work should aim to develop standardised methods for assessing and adjusting for baseline variability and learning potential.

Looking forward, the advancement of microsurgical training depends on the development of more standardised and objective evaluation tools (Hu et al., 2025). Integration of technology such as motion tracking, machine learning (ML)-assisted feedback, and immersive training platforms are increasingly explored for more precise and objective assessments. ML-assisted feedback implemented in a virtual reality setting has been shown to improve skill acquisition and provide structured guidance (Giglio et al., 2025). Such approaches may also help identify subtle technical errors and provide structured guidance, helping trainees refine their technique even in self-directed practice (Carciumaru et al., 2025). Automated tracking of skill progression over time could also help address the limitation of short-term assessment by offering insights into long-term skill retention. Incorporating such innovations into structured microsurgical training programs could overcome current limitations of short-term assessment, improve learning efficiency and retention, and ultimately accelerate the development of proficient microsurgeons.

## 5 Conclusion

In this study, the step I and step II EANS courses for microneurosurgery resulted in an improvement in the execution of microvascular anastomosis, with a significant reduction of most error types among participants. However, further development of microvascular anastomosis evaluation tools is necessary to improve the assessment and training of these skills. Future research should focus on refining assessment methods that better address clinically relevant skill indicators and integrate technology, such as motion tracking and machine learning, for more objective and precise feedback.

## Declaration of generative AI and AI-assisted technologies in the writing process

During the preparation of this work the authors used ChatGPT by OpenAI in order to improve language readability. After using this tool/service, the authors reviewed and edited the content as needed and take full responsibility for the content of the publication.

## Funding sources

This research did not receive any specific grant from funding agencies in the public, commercial, or not-for-profit sectors.

## Declaration of competing interest

The authors declare that they have no known competing financial interests or personal relationships that could have appeared to influence the work reported in this paper.
